# A new termite (Isoptera, Termitidae, Syntermitinae, *Macuxitermes*) from Colombia

**DOI:** 10.3897/zookeys.587.7557

**Published:** 2016-05-10

**Authors:** Anthony C. Postle, Rudolf H. Scheffrahn

**Affiliations:** 1 P.O. Box 5473 Cairns Queensland 4870 Australia; 2 Fort Lauderdale Research and Education Center, Institute for Food and Agricultural Sciences, 3205 College Avenue, Davie, Florida 33314 USA

**Keywords:** Isoptera, Termitidae, Syntermitinae, Macuxitermes colombicus, Colombia, new species, taxonomy

## Abstract

A new species of termite, *Macuxitermes colombicus* Postle & Scheffrahn is described from soldiers and workers collected from Departamento Magdalena, Colombia. The soldier of *Macuxitermes colombicus* differs from its lone congener in having no protuberances on the head capsule.

## Introduction

The Neotropical subfamily Syntermitinae (Isoptera: Termitidae)—“the mandibulate nasutes”—is composed of 18 genera whose distribution ranges from southern Mexico to northern Argentina. The most diagnostic character of the subfamily is, as the name suggests, soldiers that possess a nasus in addition to well-developed mandibles. The component genera vary widely in the length of the nasus, with *Syntermes* spp. and *Labiotermes* spp. having the shortest and some *Rhynchotermes* spp. the longest nasus relative to head capsule proportions (Constantino and Carvalho 2011, Rocha et al. 2011, Fontes 1985), and two genera (*Macuxitermes* and *Rhynchotermes*) have species with major and minor soldiers; nevertheless, the monophyly of the subfamily is retained throughout (Inward et al. 2007, Noirot 2001).

Within the Syntermitinae, the three monotypic genera *Macuxitermes*, *Noirotitermes*, and *Acangaobitermes* form a small monophyletic group (Rocha et al. 2012). Based on original descriptions, this conclusion may seem unlikely. The soldiers of *Macuxitermes* are dimorphic, although the minor soldiers may be quite rare, while those of *Noirotitermes* and *Acangaobitermes* are monomorphic. *Noirotitermes* has prominent spine-like protuberances towards the posterior margin of the head capsule: the head capsules of *Macuxitermes* and *Acangaobitermes* lack such protuberances. *Macuxitermes* and *Acangaobitermes* soldiers possess a relatively long, slender nasus while that of *Noirotitermes* is short and broad. The pronotum of the former two genera is more-or-less saddle-shaped, yet has a clover-like appearance in *Noirotitermes*. Furthermore, the lateral margins of the thoracic nota of both major and minor *Macuxitermes* soldiers are adorned with short dark, stout spines which are absent in *Noirotitermes* and *Acangaobitermes*. Nevertheless, the group is distinguished by having soldiers with characteristic minute granulations on the surface of the head capsule, which are absent in all other Syntermitinae, and slender, sickle-shaped, piercing mandibles with a sharply pointed, marginal tooth.

There are also differences between the workers. The morphology of the mandibles—including the absence of ridges on the molar plates—and relative dimensions of the digestive tube are essentially the same among the three taxa; however, *Macuxitermes* workers are large and robust, while those of the other two genera are small, slender, and elongate. Only *Macuxitermes* workers have notal spines (similar to those of the soldiers). In all three genera, the digestive tube displays the complete dorsal torsion as defined by Noirot (2001), while the components are of very similar disposition and relative proportions. However, although the enteric valves share an almost identical shape, the arrangement and structure of the spines in *Macuxitermes* differs from that of *Noirotitermes* and *Acangaobitermes*.

Genera of Syntermitinae exhibit a wide variety of nest-building behaviour (Constantino 1991, Emerson 1952, Emerson and Banks 1965, Fontes 1985, Redford 1984, Scheffrahn 2010, Snyder 1922). Some build epigeal mounds (*Cornitermes* and *Embiratermes* spp.) or arboreal carton nests in rotten wood or tree stumps (*Labiotermes* spp., *Silvestritermes holmgreni* (Snyder)), while some, including species of *Cyrilliotermes* and *Curvitermes*, live in abandoned nests of other termite species or as inquilines. *Labiotermes longilabius* (Silvestri) builds “underground nests lined with blackish carton” (Silvestri 1903, cited in Emerson and Banks 1965) or “ladder” nests up the sides of trees. Other species construct diffuse underground nests and galleries e.g. *Rhynchotermes bulbinasus* Scheffrahn. The *Macuxitermes* group seems to fall into the latter category, although Rocha et al. (2012) suggest that these may also be mound inquilines of other species.

## Material and methods

Specimens of *Macuxitermes colombicus* sp. n. were collected in Departamento Magdalena, Colombia, on 3 JUN 2009. Images of preserved specimens in 85% ethanol were made using an Olympus SZX9 stereomicroscope fitted with a LM Scope camera tube to an Olympus E-410 digital camera. Specimens were suspended in Purell® Instant Hand Sanitizer for transparent posturing support during photography. Enteric valve slide images were taken with an Olympus BH-2 compound microscope fitted with phase contrast optics. The entire worker P2 region was removed by micro-dissection and external muscle detached. Food particles were removed from enteric valve armature using an ultrasonic cleaner. The cleaned enteric valve was longitudinally cut, splayed open, and mounted on a microscope slide using PVA medium (BioQuip Products Inc.). External morphological terminology follows that of Roonwal (1969) and internal anatomical terminology that of Noirot (2001).

## Taxonomic treatment

### Macuxitermes

Taxon classificationAnimaliaIsopteraTermitidae

Cancello & Bandeira

#### Description.

The genus *Macuxitermes* was erected for a single species, *Macuxitermes triceratops* Cancello & Bandeira, 1992. Their generic description is modified below to include *Macuxitermes colombicus*.

**Soldier.** Dimorphic or monomorphic.

**Major soldier.** Head capsule evenly rounded or with a dorsal elevation and anterior protuberance either side of the midline, entire surface covered with minute granulations. Anterior of head capsule narrows to form a robust conical nasus whose apex extends far beyond tips of mandibles; nasus well separated from mandibles in lateral view, conical, gradually tapering to apex; without setae but terminating in a circular fontanelle, the opening of which is surrounded by numerous short hairs; mandibles curved strongly inwards, apices directed laterally at rest and not upturned, each mandible with a pointed marginal tooth half-way along inner surface; width of labrum greater than length; antennae yellow with 15 articles, I largest and III shortest; rows of dark short, tooth-like spines along the margins of the thoracic nota.

**Minor soldier.** Known only in *Macuxitermes triceratops*. See Cancello and Bandeira (1992) for description.

**Worker.** Detailed description in Cancello and Bandeira (1992) is congruent with *Macuxitermes colombicus*.

*Addenda*: left mandible with all three marginal teeth clearly visible, molar plates with no ridges; digestive tube showing complete dorsal torsion and with small crop, long and inflated mixed segment that is proximally narrow and broadly oval distally, very large and voluminous P1 and relatively smaller P3 (with diverticulum), long P4 and large P5; two pairs of Malpighian tubules, each pair joining alimentary canal separately at junction of midgut and hindgut; enteric valve narrow, on the left posterior side of the abdomen, with three finger-like cushions each bearing two rows of regularly spaced long, narrow, straight or slightly curved spines of equal length throughout; ridges separated by pads composed of a single layer of squamous cells, each with a central, slightly raised spine marginally shorter in length than spines on cushion.

### Macuxitermes colombicus

Taxon classificationAnimaliaIsopteraTermitidae

Postle & Scheffrahn sp. n.

http://zoobank.org/6F05594E-14EB-4762-9E90-B3A16D7D32B8

Figs 1 , 2 , 3 , 4 , 5 , 6 , 7 , 8

#### Material examined.

Holotype. Soldier. Labelled “(UF code CO442) Colombia, Depto. Magdalena, Ciénaga de Ortiz, 10.15187 –75.04366, 3JUN2009, col. SBCMKSN”. The holotype is kept in the same vial as the paratypes.

#### Type locality.

COLOMBIA. Elevation 44 m

#### Paratypes.

One soldier and 12 workers. The material examined was hand-collected by John R. Mangold. The type and paratypes are deposited in the University of Florida Termite Collection (Ft. Lauderdale Research and Education Center, Davie, Florida).

#### Etymology.

The species name is derived from the latinization of Colombia, the type country.

#### Habitat and biology

**(Fig. 8).** Foragers were collected under a tree branch that had recently fallen in a cattle pasture. The wood had not been attacked, suggesting that this termite is probably a soil feeder.

#### Description.

**Winged imago.** unknown.

**Minor soldier.** unknown.

**Figure 1.**
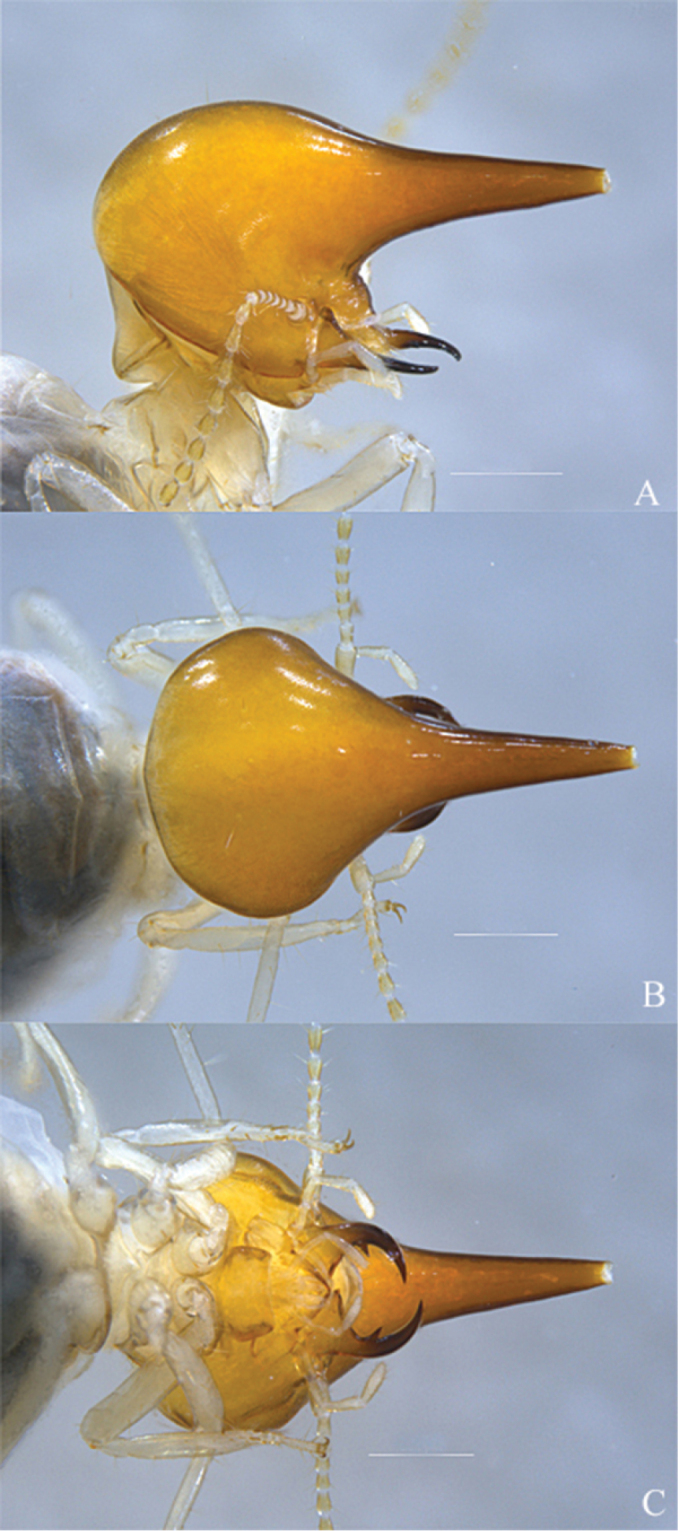
*Macuxitermes colombicus* soldier. **A** lateral **B** dorsal and **C** ventral views of head. Scale: 500 μm.

**Figure 2.**
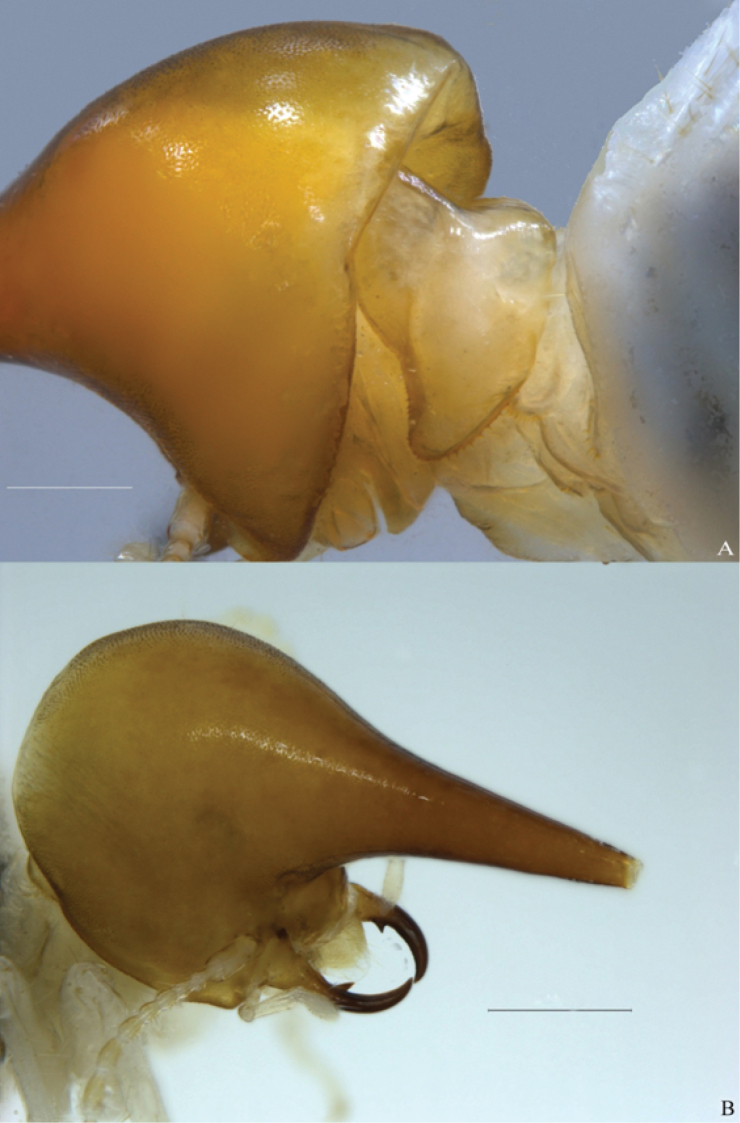
*Macuxitermes colombicus* soldier. **A** posterolateral and **B** anterolateral views of head capsule showing surface pitting. Scale: 500 µm.

**Major soldier (Figs 1, 2, 8).** Monomorphic. Head capsule with characteristic microsculpture and with a few long, pale setae on posterior margin; in profile, nasus at an angle of 45 degrees to inferior angle of head capsule but slightly convergent with plane of mandibles; fronto-clypeal region slightly inflated, length less than one-third width, distal margin hyaline, postclypeus with transverse rugulae; labrum broader than long, with convex sides and rounded apex, distal margin hyaline; postmentum very short; mandibles not upturned apically, marginal teeth tapering distally to a sharp point which is directed anteriorly; antennae yellow with 15 articles, 1>2>3=4=5.

Pronotum narrower than head, anterior lobe longer than broad, arising very steeply from posterior lobe so that it is positioned like a brace under the posterior margin of the head capsule, posterior lobe more than twice as broad as long, lateral and postero-lateral margins with numerous dark short, tooth-like spines; lateral margins of meso- and metanotum with similar spines. Legs slender with irregularly spaced long, pale, fine, erect setae on femora, tibiae and tarsi, fore-coxae ridged but with no projecting keel, fore-tibiae slightly inflated, fore- and mid-coxae with two short, dark, stout distal spines on inner ventral surface near junction with trochanter; tibial spur formula 2: 2: 2. Abdominal tergites and sternites with numerous, closely packed, long and short pale, erect setae.

Measurements – mean and range in mm (n=2): head length with nasus: 2.30 (2.20–2.40), head length to base of mandibles: 0.96 (no range), maximum head width: 1.36 (1.32–1.40), maximum pronotal width: 0.82 (0.80–0.84), length of hind tibia: 1.17 (1.16–1.18).

**Figure 3.**
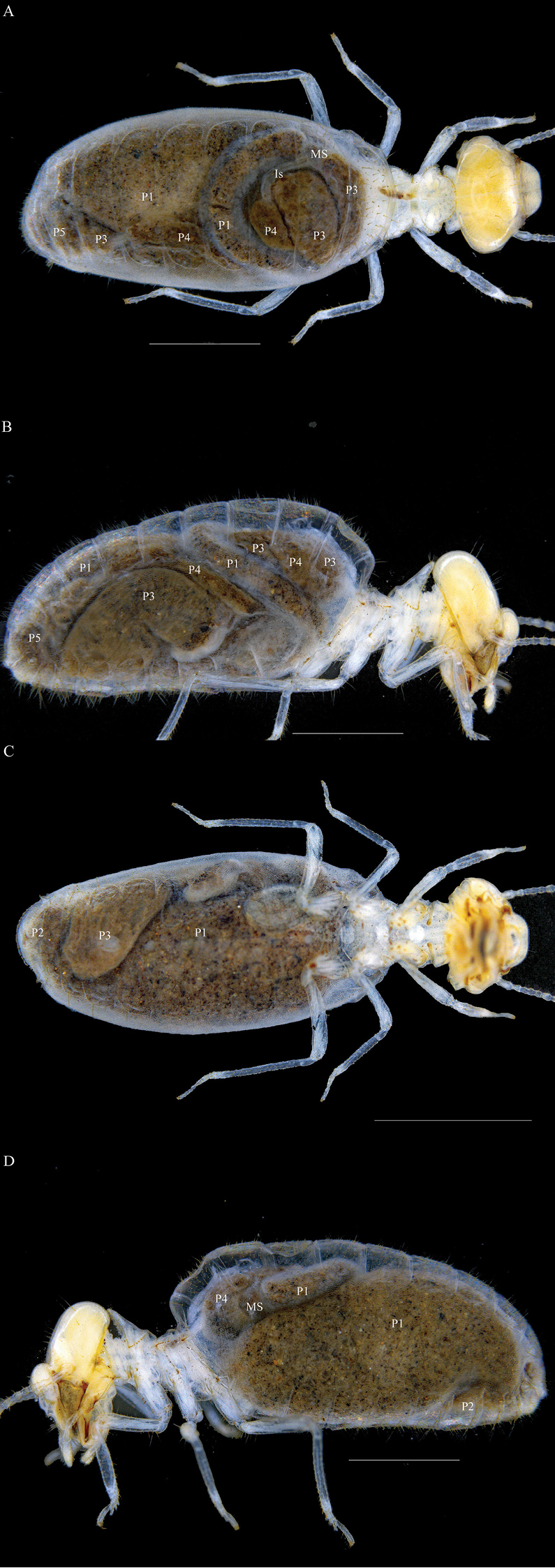
*Macuxitermes colombicus* worker. **A** dorsal **B** lateral (right) **C** ventral, and **D** lateral (left) views. Is, Isthmus; MS, Mixed segment, P1, P2, P3, P4 and P5 proctodeal segments 1-5, respectively. Scale: 1mm.

**Figure 4.**
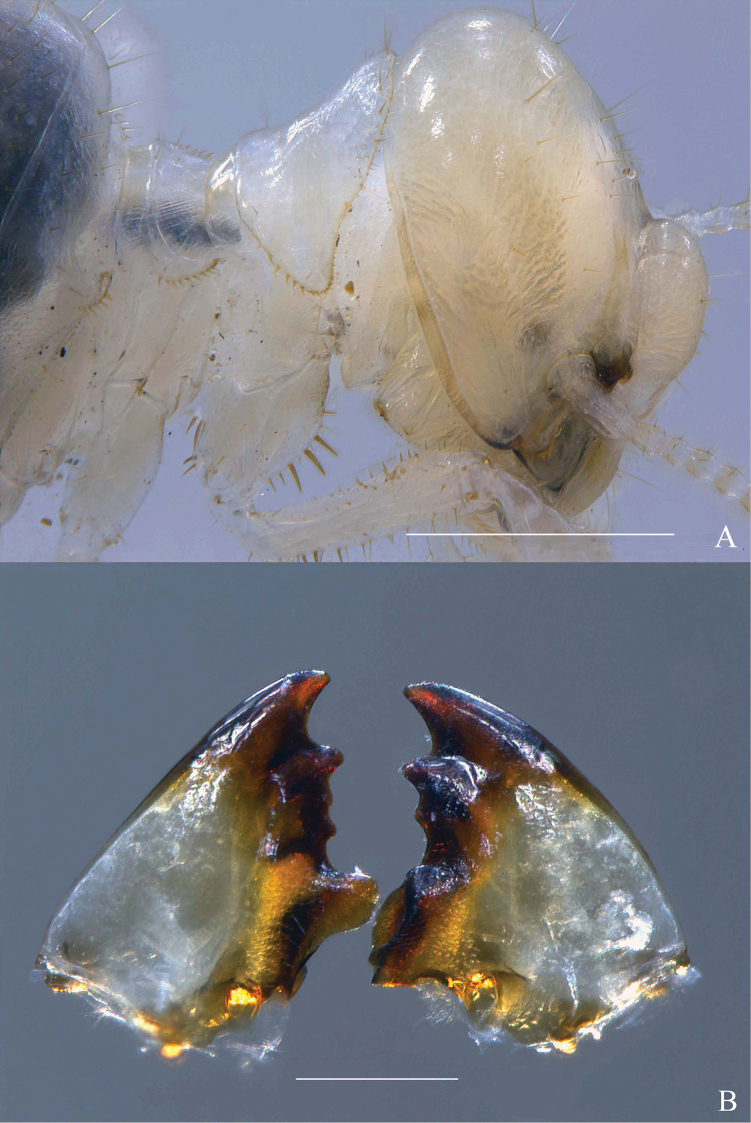
*Macuxitermes colombicus* worker. **A** head and thorax **B** mandibles (somewhat worn). Scale: 500 μm (**A**), 200 μm (**B**).

**Figure 5.**
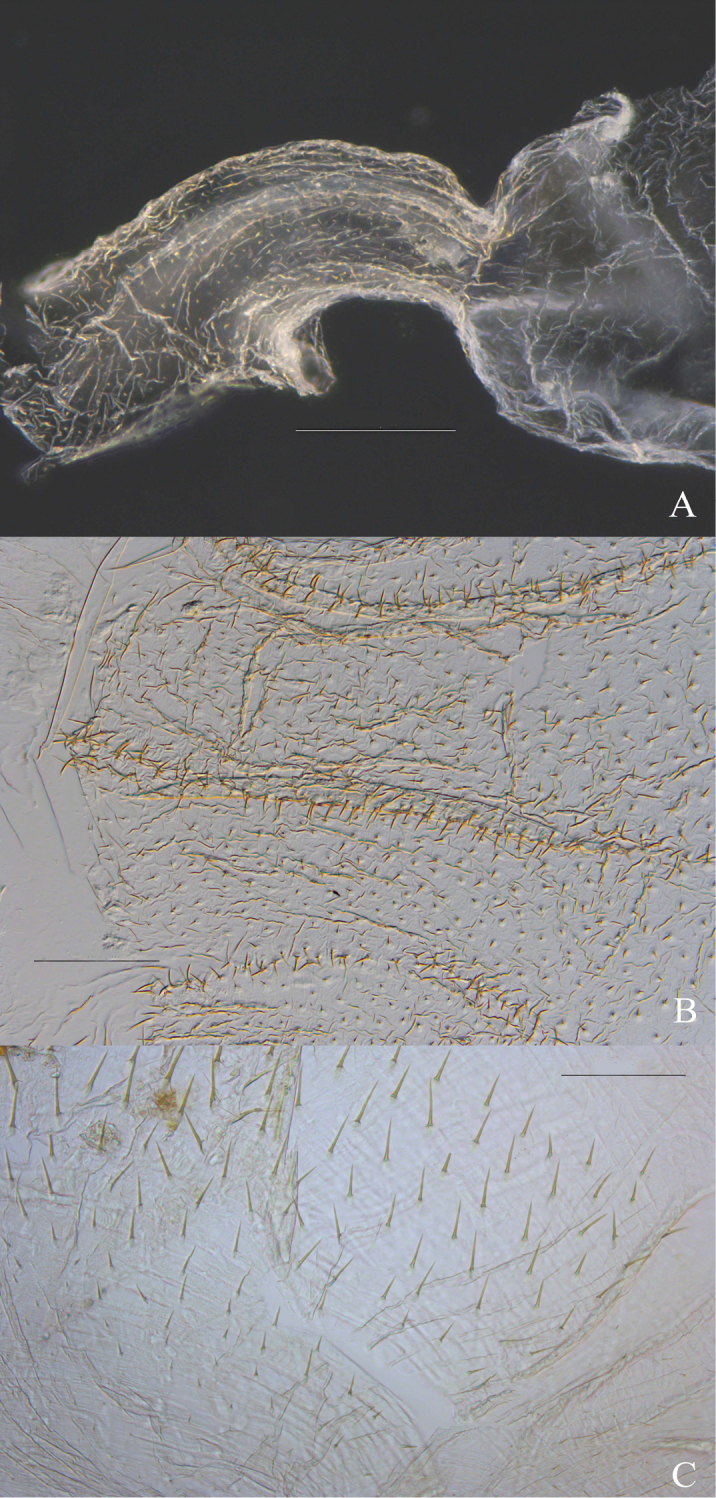
*Macuxitermes colombicus* worker. **A** P2 and connection with P3 **B** enteric valve ridges, and **C** aciculiform spines near junction of P1 and mixed segment. Scale: 200 μm (**A**), 100 μm (**B, C**)

**Figure 6.**
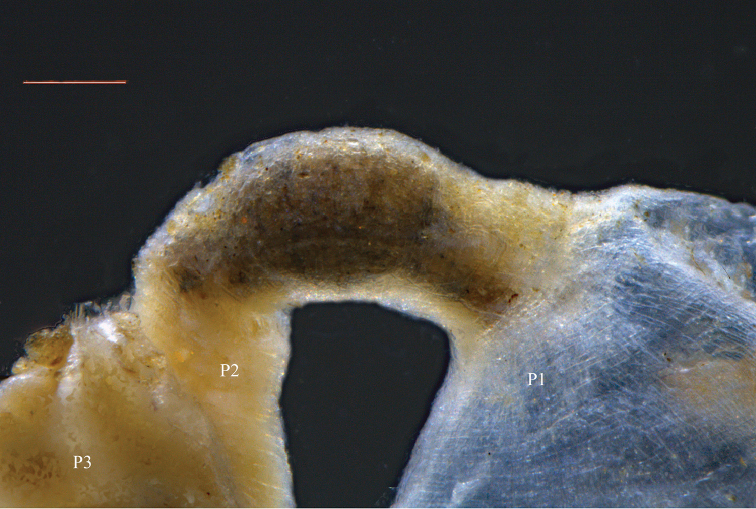
*Macuxitermes colombicus* worker alimentary canal showing P2 at junction of P1 and P3. Scale: 100 µm.

**Figure 7.**
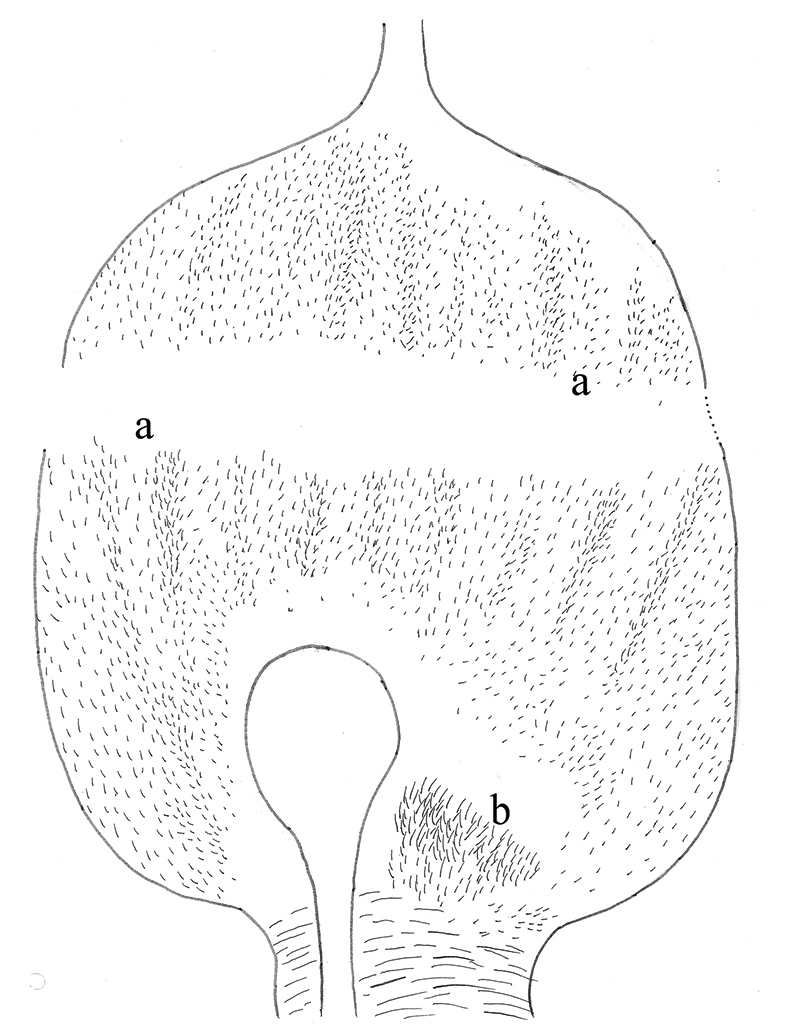
*Macuxitermes colombicus* worker alimentary canal: schematic drawing of P1 showing position and arrangement of **a** short inner-surface spines and **b** aciculiform spines.

**Figure 8.**
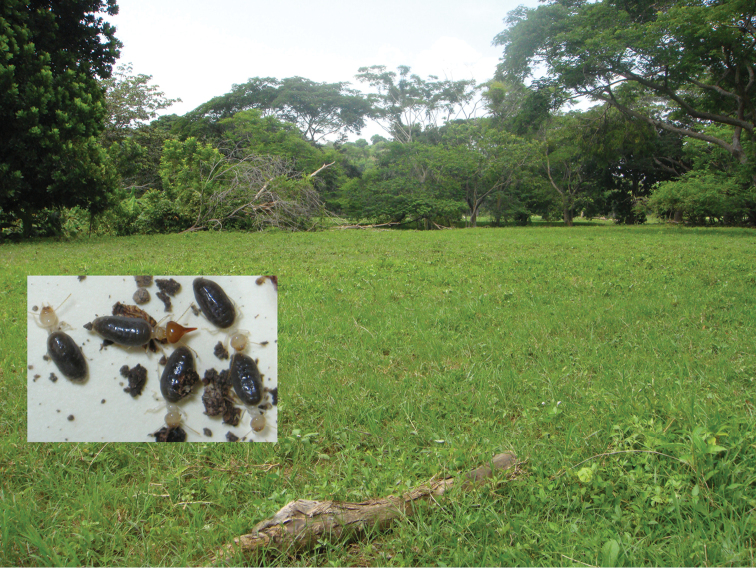
*Macuxitermes colombicus* type locality and live workers and soldier habitus (inset).

**Worker (Figs 3–8).** Monomorphic. Body slightly smaller than that of the soldier. Fig. 8 shows workers and soldier to have exceptionally contrasting coloration between the abdomen (very dark) and thorax (very pale). Head rounded, with scattered long, pale, fine, erect setae; postclypeus strongly inflated, labrum with two long erect setae either side of midline; both mandibles with apical tooth longer than marginal teeth, inner margin of each apical tooth slightly concave and longer than the anterior margin of the first marginal tooth, second marginal tooth small but distinct, molar plates with no obvious ridges; left mandible with posterior margin of first marginal tooth slightly concave, second marginal tooth slightly smaller than and well separated from third marginal tooth, molar plate clearly visible although covered by molar process which projects beyond apex of third marginal tooth; right mandible with second marginal tooth much smaller than first but evident, posterior margin of first marginal tooth concave, posterior margin of second marginal tooth concave to molar plate which is very reduced and anterior to molar process; antennae with 15 articles, basal articles pale yellow, distal five-seven articles darker yellow.

Pronotum narrower than head, anterior lobe as in soldier, much longer than broad and rising at a very steep angle (>80 degrees) from the posterior lobe, about as long as broad; anterior margin with a row of long, pale, erect setae interspersed with smaller setae, posterior lobe short and broad; lateral and postero-lateral margins of pronotum and lateral margins of meso- and metanotum with numerous short, dark, serrations or tooth-like spines. Legs slender with numerous long, pale, erect setae on femora, tibiae and tarsi, a few prominent dark spines on anterior and inner ventral surfaces of fore-coxae and basal region of fore-femora, shorter, irregularly spaced shorter dark spines elsewhere on these segments, fore-coxae ridged but with no projecting keel, fore-tibiae slightly inflated, a ventral row of longer dark spines, along with a few scattered, much shorter dark spines on distal half; tibial spur formula 2: 2: 2. Tergites and sternites with numerous closely packed, long and short pale, fine, erect setae.

Digestive tube almost identical to that of *Macuxitermes triceratops* as depicted in Constantino (1997) (See generic description for details). The cross-section of the gizzard resembles very closely that of *Cornitermes cumulans* (Kollar) as illustrated in Noirot (2001), in possessing a well-sclerotized columnar belt and a lightly sclerotized pulvillar belt. The pulvilli appear to lack spines. The internal ornamentation of P1 is similar to that of *Macuxitermes triceratops* as illustrated by Rocha and Constantini (2015) but the arrangement of the proximal aciculiform spines is different.

Measurements – mean and range in mm (n=12): head length with nasus/mandibles: 1.34 (1.28–1.44), head length to base of mandibles: 0.86 (0.84–0.92), maximum head width: 1.09 (1.04–1.12), maximum pronotal width: 0.62 (0.56–0.64), length of hind tibia: 1.06 (1.04–1.12).

## Discussion

The new species was assigned to *Macuxitermes* after careful assessment and consideration of the morphological and anatomical data that have been assembled and reported on the component genera of Syntermitinae. Like *Macuxitermes triceratops*, the head capsule of the soldier is endowed with fine microsculpture on the surface and the soldier and worker castes have notal spines, while the mandibles, mesenteric tongue and enteric valve of the workers match the descriptions of these structures in this species. However, several of these features are also found in members of the genus *Armitermes* *s. s.*. Our species does in fact resemble *Armitermes* and differs conspicuously from *Macuxitermes triceratops* in its appearance. The mandibles in the major soldier are less robust than those of *Macuxitermes triceratops* and the postmentum, although very short, is not noticeably inflated. The profile of the nasus is straight rather than curved and the head capsule lacks the anterior processes of *Macuxitermes triceratops* plus the occipital protuberances which are a feature of other members of the *Macuxitermes* group (Rocha et al. 2012). Furthermore, *Macuxitermes colombicus* might not have minor soldiers.

Nevertheless, this termite does not fit readily into *Armitermes* either. The shape of the head capsule of the soldier differs from those of the three known species of *Armitermes*; nor is the minute pitting that covers its surface a characteristic of the latter. The labrum of *Macuxitermes colombicus* soldiers is broadly rounded, while the mandibles have a distinctly different configuration from those of *Armitermes* spp.. As stated, *Macuxitermes* does share with *Armitermes* *s. s.* the presence of notal spines in both soldiers and workers but this is not considered evidence of close relationship (Constantino 1997, Rocha et al. 2012). In addition, the notal spines on the new species are far more numerous and extensive than in *Armitermes* and the bristles of the pro-, meso- and metanotum of the soldier, reported by Rocha et al. (2012) as definitive of this genus, are lacking. In the worker, there is a greater degree of pilosity and the enteric valve is different from the type diagnostic of *Armitermes* spp.

Rather than create a new genus, the authors therefore place this species in *Macuxitermes* to which it seems to have the greatest affinity. As well as the previously listed similarities, it has the following in common with *Macuxitermes triceratops*. Although worn down to some degree in all specimens examined, the second marginal tooth in the worker is reduced but distinct on both mandibles. Thus, all three marginal teeth are clearly visible on the left mandible. For consistency, the term “first-plus-second marginal tooth” should perhaps be retained for the left mandible, although the degree of fusion seems much less than in other genera and species of the sub-family.

The enteric valve of the worker very closely resembles that of *Macuxitermes triceratops*. It is also very similar in appearance and constitution to those of *Noirotitermes*, *Acangaobitermes*, *Embiratermes*, *Ibitermes*, and *Uncitermes* (Constantino 1997, Cancello and Myles 2000, Rocha et al. 2012). However, it differs from these in having spines of equal length on the finger-like ridges, rather than spines that increase in length distally. The ridges in *Ibitermes* are also slightly dilated apically. Thus, the composition of the enteric valve may also be a diagnostic feature of *Macuxitermes*.

The absence of pulvillar spines as a generic feature is yet to be determined. Although pulvilli are described as “lacking armature or ornamentation” in *Acangaobitermes* (Rocha et al., 2011), the presence or absence of such is not reported for *Macuxitermes triceratops* (Constantino 1997) or *Noirotitermes* (Cancello & Myles, 2000). Because of the apparent close similarity in appearance between the structure of the gizzard in *Macuxitermes colombicus* and *Cornitermes cumulans*, it would be of interest if further studies could reveal the degree of uniformity of the gizzard’s musculature and cuticular armature throughout the sub-family, however.

It is possible that DNA analyses may provide greater insight into the relationship of *Macuxitermes colombicus* with its congener. It is also possible that future field studies may yield further specimens of the species, including minor soldiers, thereby confirming its current taxonomic status. Until then, its placement in the genus *Macuxitermes* is in concordance with the available data.

## Supplementary Material

XML Treatment for Macuxitermes

XML Treatment for Macuxitermes colombicus
